# Sagittal alignment changes predict patellar osteosclerosis after total knee arthroplasty without resurfacing

**DOI:** 10.1002/jeo2.70750

**Published:** 2026-05-22

**Authors:** Shunsuke Utsumi, Takashi Aki, Yu Mori, Masayuki Kamimura, Kento Harada, Hiroaki Sato, Tomokazu Tanita, Toshiya Uehara, Toshimi Aizawa

**Affiliations:** ^1^ Department of Orthopaedic Surgery Tohoku University Graduate School of Medicine Sendai Japan

**Keywords:** femorotibial posterior overhang, patellar osteosclerosis, patellofemoral joint, posterior tibial slope, total knee arthroplasty

## Abstract

**Purpose:**

Total knee arthroplasty (TKA) is an effective treatment for knee osteoarthritis; however, some patients experience post‐operative anterior knee pain related to patellofemoral (PF) loading. Subchondral patellar osteosclerosis on radiographs may indirectly reflect increased PF stress. This study examined whether changes in posterior tibial slope (PTS) and femorotibial posterior overhang (FTPO) are associated with patellar osteosclerosis after TKA without patellar resurfacing.

**Methods:**

A total of 94 knees (71 patients) that underwent TKA without patellar resurfacing for medial OA using the Journey II Bi‐Cruciate Stabilized (BCS) system were retrospectively reviewed. Knees were classified according to the presence of post‐operative patellar osteosclerosis. Group differences were evaluated using the Mann–Whitney *U* test, and correlations between ΔPTS and ΔFTPO were assessed with Spearman's coefficient. Receiver operating characteristic (ROC) analysis and multivariable logistic regression were performed to determine cut‐off values and independent predictors.

**Results:**

Patellar osteosclerosis developed in 26 out of 94 knees (27.7%). The osteosclerosis group showed significantly greater post‐operative reductions in both PTS and FTPO (*p* < 0.001). ΔPTS and ΔFTPO were positively correlated (*r* = 0.35, *p* = 0.0006). ROC analysis identified cut‐offs of ΔPTS ≤ −0.28° (AUC = 0.91) and ΔFTPO ≤ 0.68 mm (AUC = 0.84). Multivariable analysis confirmed that more negative ΔPTS (odds ratio [OR] = 0.49, 95% confidence interval [CI] = 0.34–0.71) and smaller ΔFTPO (OR = 0.78, 95% CI = 0.65–0.93) independently predicted patellar osteosclerosis.

**Conclusion:**

Post‐operative reductions in PTS and relative femoral anteriorization were associated with patellar osteosclerosis after TKA without patellar resurfacing. These sagittal alignment changes may contribute to increased PF joint stress and anterior knee pain. Although PF pressure and symptoms were not directly evaluated, avoiding excessive PTS reduction and femoral anteriorization may help minimize PF overloading.

**Level of Evidence:**

Level III.

## Graphical abstract

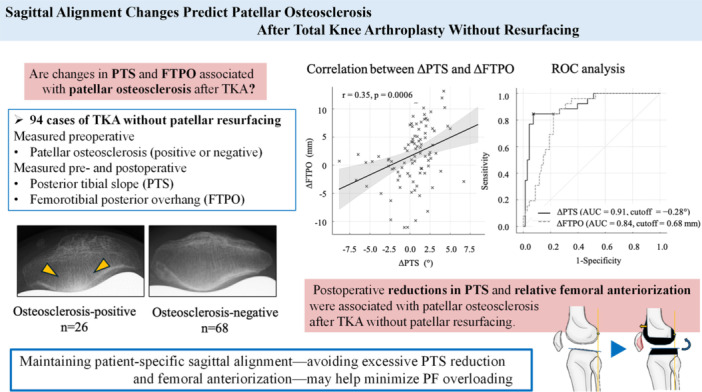

**Abbreviations**
AUC: area under the curve
BCS: bi‐cruciate stabilized
CI: confidence interval
FTPO: femorotibial posterior overhang
NOS: non‐osteosclerosis
NPV: negative predictive value
OA: osteoarthritis
OS: osteosclerosis
PCA: posterior condylar axis
PF: patellofemoral
PFA: patellar facet angle
PPV: positive predictive value
PTS: posterior tibial slope
ROC: receiver operating characteristic
SEA: surgical epicondylar axis
TKA: total knee arthroplasty
VIF: variance inflation factor

## INTRODUCTION

Total knee arthroplasty (TKA) is a well‐established and effective treatment for osteoarthritis (OA) of the knee, with both short‐ and long‐term outcomes having improved substantially owing to advances in implant design, surgical techniques and computer‐assisted technologies [2, 4, 9]. Nevertheless, a subset of patients continues to experience unsatisfactory results after TKA, and anterior knee pain related to the patellofemoral (PF) joint remains one of the major causes of post‐operative dissatisfaction [18].

Elevated PF joint pressure has been suggested as a potential factor contributing to post‐operative anterior knee pain [18, 21]. In TKA without patellar resurfacing, subchondral osteosclerosis on post‐operative radiographs may appear as a manifestation of increased PF loading and has been used as an indirect indicator of elevated PF joint stress [20, 23]. Multiple factors influence PF joint pressure, including femoral component rotational alignment, trochlear groove geometry, and patellar morphology and thickness of the patella [3, 7, 12]. In the sagittal plane, posterior tibial slope (PTS) [6] and the anteroposterior positional relationship between the femur and tibia also play important roles in PF joint mechanics [10].

Prior computational studies demonstrated that a decrease in PTS increases PF contact pressure following TKA [16]. Furthermore, anterior positioning of the femur relative to the tibia may elevate PF stress. Femorotibial posterior overhang (FTPO) has recently been proposed as an index of sagittal femorotibial anteroposterior alignment [14]. FTPO reflects the degree of femoral anteriorization after TKA and has been suggested to influence PF kinematics. However, the combined influence of the changes in PTS and FTPO on PF pressure after TKA remains poorly understood. Although previous studies have independently examined PTS and AP alignment parameters, no study has evaluated their combined effect on PF loading or patellar osteosclerosis.

Therefore, the purpose of this study was to investigate the association between pre‐ and post‐operative changes in PTS and FTPO and the occurrence of patellar osteosclerosis following TKA without patellar resurfacing, to clarify the potential impact of sagittal alignment changes on PF joint stress. Understanding these sagittal alignment factors may help optimize surgical planning and reduce post‐operative PF‐related symptoms. We hypothesized that a reduction in PTS and anterior translation (reflected by decreased FTPO) of the femur would be associated with the development of post‐operative patellar osteosclerosis.

## MATERIALS AND METHODS

### Study design and patient selection

This retrospective study included 94 knees in 71 patients who underwent TKA without patellar resurfacing for medial OA at our institution between January 2017 and May 2024. The cohort consisted of 16 males and 55 females, with a mean age of 75 years (range, 58–84 years). All procedures used the Journey II Bi‐Cruciate Stabilized (BCS) system (Smith & Nephew, Inc.). Patellar non‐resurfacing was indicated based on the patellar facet angle (PFA) measured on preoperative skyline radiographs. Following the criteria described by Takahashi et al. [18], knees with PFA ≥ 132° were selected for non‐resurfacing.

Exclusion criteria included patients with preoperative patellar osteosclerosis, severe PF OA with obliterated joint space or patellar deformity, PF‐related symptoms such as anterior knee pain, lateral compartment OA, post‐traumatic OA and rheumatoid arthritis.

### Surgical procedure

All procedures were performed using a medial parapatellar approach. Coronal alignment targeted a mechanical axis perpendicular to the femoral and tibial mechanical axes. Sagittal alignment targeted perpendicularity to the distal femoral anatomical axis and a PTS of 4°.

The surgical epicondylar axis (SEA) was defined as the target reference for femoral rotational alignment. To improve the accuracy of SEA identification, the angular difference between the SEA and the posterior condylar axis (PCA) was calculated preoperatively on CT images for each individual case. Intraoperatively, a posterior referencing guide based on the PCA was used to reproduce the planned rotational alignment targeting the SEA as accurately as possible.

Concurrently, dynamic assessment using a modified gap technique was performed [13]. Specifically, gap measurements were obtained under 60 N of distraction force using a tensor device, and femoral rotational alignment was fine‐tuned according to the difference between the lateral and medial flexion gaps. Tibial rotation was referenced to Akagi's Line [1].

### Radiographic measurements

**Figure 1:**
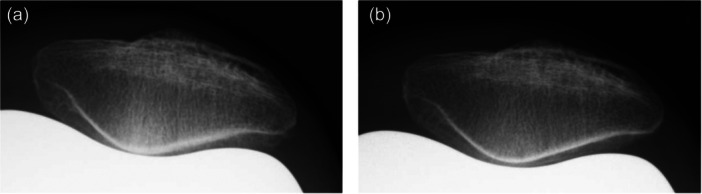
Post‐operative axial radiographs of the patella obtained in 45° of knee flexion under non‐weight‐bearing conditions, 1 year after TKA. (a) A representative case with patellar osteosclerosis (patellar osteosclerosis‐positive), showing increased subchondral bone density in the patellofemoral joint. (b) A representative case without patellar osteosclerosis (patellar osteosclerosis‐negative). TKA, total knee arthroplasty.

**Figure 2:**
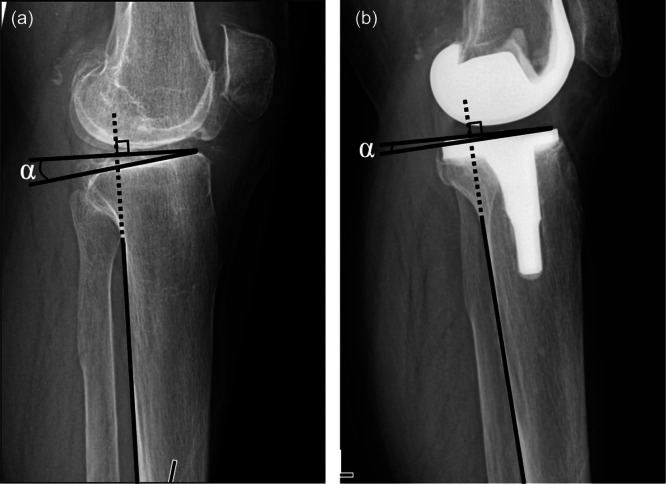
Measurement of preoperative and post‐operative posterior tibial slope (PTS). PTS was measured on lateral knee radiographs obtained before and after surgery. PTS was calculated from the angle between the proximal tibial joint surface and the posterior tibial cortex. (a) Preoperative PTS was defined as *α*, and (b) post‐operative PTS was defined as *α* + 4°, including the insert slope.

**Figure 3:**
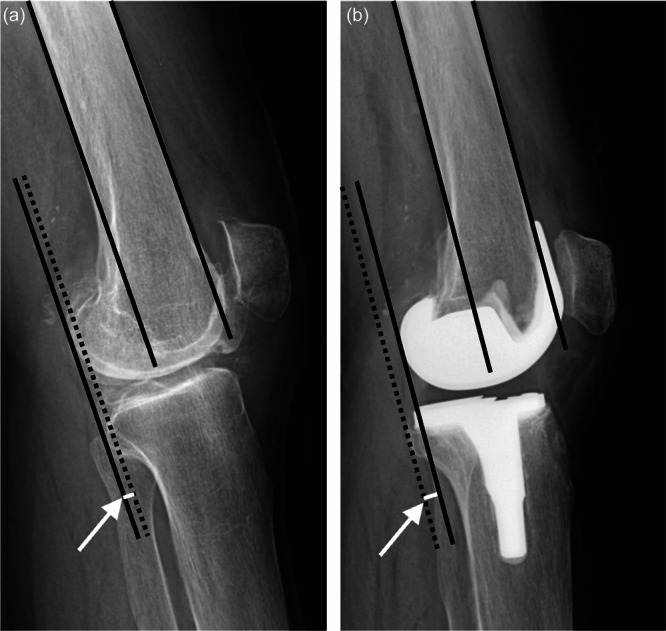
Measurement of preoperative and post‐operative femorotibial posterior overhang (FTPO). FTPO was assessed on lateral knee radiographs obtained before and after surgery. FTPO is an indicator of the relative sagittal position of the femur with respect to the tibia and was defined as the distance between the solid line representing the femur and the dotted line representing the tibia. (a) A representative preoperative x‐ray showing a positive FTPO value, which indicates posterior femoral overhang relative to the tibia. (b) A representative post‐operative x‐ray showing a negative FTPO value, which indicates anterior femoral positioning relative to the tibia.

PFA was measured using preoperative skyline radiographs. At 1 year post‐operatively, subchondral patellar osteosclerosis was evaluated using skyline radiographs. It was strictly defined as newly developed subchondral sclerosis—specifically, a localized decrease in radiolucency or an increase in bone density at the patellar articular surface (Figure 1). The reliability of this radiographic assessment was robust, demonstrating an intra‐observer kappa coefficient of 0.74 and an inter‐observer kappa of 0.73. To accurately evaluate the post‐operative development of this condition, cases exhibiting patellar osteosclerosis on preoperative radiographs were rigorously excluded from the study cohort. PTS and FTPO were evaluated on lateral radiographs in full extension preoperatively and at 1 year post‐operatively. PTS was measured as the angle between the tibial plateau and posterior tibial cortex (Figure 2) [5], and post‐operative PTS was corrected by adding the 4° insert angle. FTPO was assessed according to Murata et al. [14], positive values indicating posterior femoral overhang and negative values indicating anterior femoral positioning relative to the tibia (Figure 3). Changes in PTS and FTPO (ΔPTS, ΔFTPO) were calculated as post‐operative minus preoperative values. Negative ΔPTS indicated a post‐operative reduction, whereas negative ΔFTPO indicated anterior translation of the femur. Based on the presence or absence of post‐operative patellar osteosclerosis, knees were classified into an osteosclerosis (OS) group and a non‐osteosclerosis (NOS) group for subsequent comparative analysis.

### Statistical analysis

Interobserver and intraobserver reliabilities for PTS and FTPO measurements were evaluated using the intraclass correlation coefficient [ICC (2,1)] with a two‐way random‐effects model (absolute agreement). Interobserver ICCs ranged from 0.83 to 0.96, indicating good to excellent reliability, and intraobserver ICCs ranged from 0.95 to 0.99, indicating excellent reliability. Categorical variables were analyzed using the chi‐square test. Continuous variables, including PFA, PTS, FTPO and their changes (ΔPTS, ΔFTPO), were compared between the OS group and NOS group using the Mann–Whitney *U* test. Correlations between ΔPTS and ΔFTPO were assessed using Spearman's rank correlation coefficient. Receiver operating characteristic (ROC) curves with the Youden index were used to identify cut‐off values, and the area under the curve (AUC) was calculated using 1000‐iteration bootstrap resampling. Multivariable logistic regression was performed to identify independent predictors of patellar osteosclerosis, including ΔPTS, ΔFTPO, age and sex. Multicollinearity was evaluated using correlation matrices and the variance inflation factor (VIF). Sensitivity, specificity, positive predictive value (PPV) and negative predictive value (NPV) with 95% confidence intervals (CIs) were calculated for each cut‐off value. Combined prediction models using ‘AND’ and ‘OR’ rules were also assessed. Statistical analyses were set at *p* < 0.05. Analyses were performed using JMP version 18 (SAS Institute).

## RESULTS

Among 94 knees included in this study, 26 (27.7%) exhibited patellar osteosclerosis at 1 year post‐operatively, while 68 knees (72.3%) did not. There were no significant differences in age or sex distribution between the OS and NOS groups. Preoperative PFA was also comparable between groups (OS: 137.3 ± 4.9°; NOS: 137.4 ± 4.1°) (Table 1).

**Table 1:** Comparison of patient demographics and preoperative clinical characteristics according to the presence of post‐operative patellar osteosclerosis.

Demographics	OS group (*n* = 26)	NOS group (*n* = 68)	*p*
Mean age (y/o) ± SD	76.5 ± 5.4	75.4 ± 6.0	0.37
Male patient (%)	8 (30.8%)	11 (16.2%)	0.12
Mean PFA (°) ± SD	137.3 ± 4.9	137.4 ± 4.1	0.44

Abbreviations: NOS, osteosclerosis‐negative; OS, osteosclerosis‐positive; PFA, patellar facet angle; SD, standard deviation.

### PTS

Overall, the mean PTS was 4.4 ± 2.8° preoperatively and 4.8 ± 1.9° post‐operatively. Preoperative PTS was significantly greater in the OS group than in the NOS group (6.6 ± 2.8° vs. 3.5 ± 2.4°, *p* < 0.001), whereas post‐operative PTS did not differ significantly between groups (4.4 ± 2.4° vs. 5.0 ± 1.7°, *p* = 0.29). The changes in PTS (ΔPTS) differed markedly between groups: PTS decreased post‐operatively in the OS group and increased in the NOS group (−2.1 ± 2.3° vs. 1.5 ± 2.2°, *p* < 0.001) (Table 2).

**Table 2:** Comparison of preoperative and post‐operative parameters in TKA.

Variable	OS group (*n* = 26), mean ± SD	NOS group (*n* = 68), mean ± SD	*p*
Pre‐operative PTS (°)	6.6 ± 2.8	3.5 ± 2.4	<0.001
Post‐operative PTS (°)	4.4 ± 2.4	5.0 ± 1.7	0.29
ΔPTS (°)	−2.1 ± 2.3	1.5 ± 2.2	<0.001
Pre‐operative FTPO (mm)	−0.8 ± 4.3	−3.9 ± 4.7	0.0039
Post‐operative FTPO (mm)	−3.0 ± 3.5	−0.4 ± 3.5	0.003
ΔFTPO (mm)	−2.2 ± 3.8	3.5 ± 4.8	<0.001

*Note*: Δ indicates the difference between post‐operative and preoperative values (Post‐op minus Pre‐op).

Abbreviations: FTPO, femorotibial posterior overhang; NOS, osteosclerosis‐negative; OS, osteosclerosis‐positive; PTS, posterior tibial slope; SD, standard deviation; TKA, total knee arthroplasty.

### FTPO

Preoperatively, the femur was positioned more posteriorly relative to the tibia in the OS group compared with the NOS group (−0.8 ± 4.3 mm vs. −3.9 ± 4.7 mm, *p* = 0.0039). Post‐operatively, the femur was positioned more anteriorly in the OS group (−3.0 ± 3.5 mm vs. −0.4 ± 3.5 mm, *p* = 0.003). Consequently, ΔFTPO was significantly smaller in the OS group than in the NOS group (−2.2 ± 3.8 mm vs. 3.5 ± 4.8 mm, *p* < 0.001), indicating greater femoral anteriorization in knees that developed patellar osteosclerosis (Table 2).

### Correlation between ΔPTS and ΔFTPO

**Figure 4:**
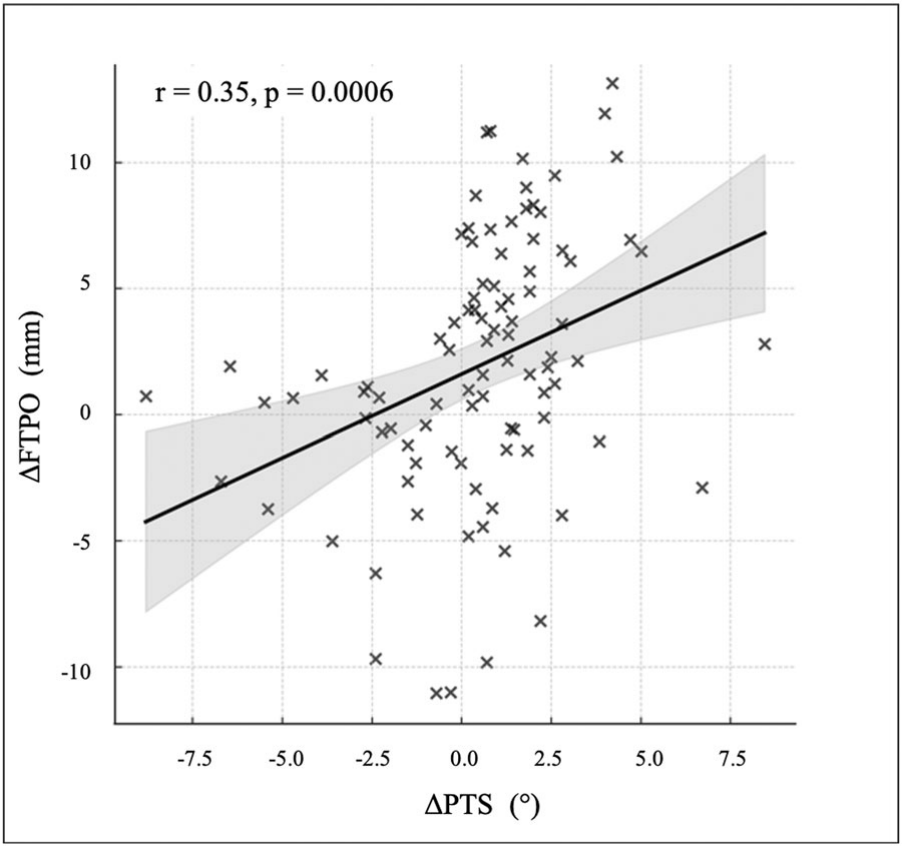
Scatter plot showing the correlation between the change in posterior tibial slope (PTS) and the change in femorotibial posterior overhang (FTPO) following total knee arthroplasty. A significant positive correlation was observed (*r* = 0.35, *p* = 0.0006).

A positive correlation was observed between ΔPTS and ΔFTPO (*r* = 0.35, *p* = 0.0006), suggesting that reductions in PTS were associated with relative anterior translation of the femur with respect to the tibia (Figure 4).

### ROC curve analysis

**Figure 5:**
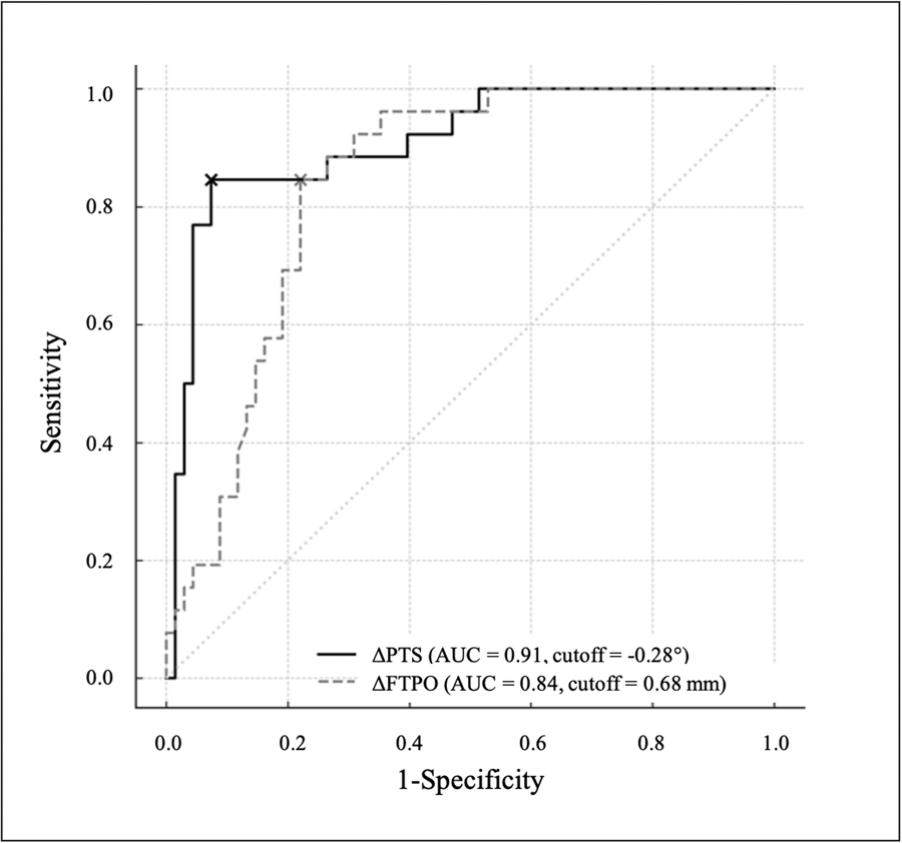
Receiver operating characteristic (ROC) curves showing the predictive ability of change in posterior tibial slope (PTS) and change in femorotibial posterior overhang (FTPO) for the presence of patellar osteosclerosis. The optimal cut‐off values determined using the Youden index were −0.28° for ΔPTS (AUC = 0.910) and 0.68 mm for ΔFTPO (AUC = 0.838).

ROC analysis demonstrated good discriminative ability of both ΔPTS and ΔFTPO for the presence of patellar osteosclerosis (Figure 5). The optimal cut‐off value for ΔPTS was ≤−0.28° (AUC = 0.910), indicating that even a small reduction in PTS was associated with osteosclerosis. For ΔFTPO, the optimal cut‐off was ≤0.68 mm (AUC = 0.838), reflecting relative anterior femoral positioning.

*Using these cut‐offs* (Table 3):

**Table 3:** Diagnostic performance of each model for predicting patellar osteosclerosis.

Model	Sensitivity (95% CI)	Specificity (95% CI)	PPV (95% CI)	NPV (95% CI)
PTS_pred	0.85 (0.67–0.94)	0.93 (0.84–0.97)	0.82 (0.63–0.92)	0.94 (0.86–0.98)
FTPO_pred	0.85 (0.67–0.94)	0.78 (0.67–0.86)	0.60 (0.44–0.74)	0.93 (0.83–0.97)
Combined_OR	0.96 (0.81–0.99)	0.72 (0.60–0.81)	0.57 (0.42–0.70)	0.98 (0.90–1.00)
Combined_AND	0.73 (0.54–0.86)	0.99 (0.92–1.00)	0.95 (0.76–0.99)	0.91 (0.82–0.95)

*Note*: Values are presented as point estimates with 95% CIs calculated using Wilson's method. PTS_pred and FTPO_pred indicate binary prediction models(1 = predicted osteosclerosis, 0 = predicted none) based on the optimal cut‐off values derived from ROC analysis. ‘Combined_OR’ represents the model in which either criterion (PTS or FTPO) is positive, whereas ‘Combined_AND’ requires both to be positive.

Abbreviations: CI, confidence interval; FTPO, femorotibial posterior overhang; NPV, negative predictive value; PPV, positive predictive value; PTS, posterior tibial slope.

ΔPTS ≤ −0.28°: sensitivity 0.85 (95% CI = 0.67–0.94), specificity 0.93 (95% CI = 0.84–0.97).

ΔFTPO ≤ 0.68 mm: sensitivity 0.85 (95% CI = 0.67–0.94), specificity 0.78 (95% CI = 0.67–0.86).

*Combined rules* (Table 3):

OR rule (ΔPTS ≤ −0.28° or ΔFTPO ≤ 0.68 mm): sensitivity 0.96 (95% CI = 0.81–0.99), specificity 0.73 (95% CI = 0.54–0.86).

AND rule (ΔPTS ≤ −0.28° and ΔFTPO ≤ 0.68 mm): sensitivity 0.73 (95% CI = 0.54–0.86), specificity 0.99 (95% CI = 0.92–1.00).

### Multivariable analysis

In multivariable logistic regression including ΔPTS, ΔFTPO, age and sex, both ΔPTS and ΔFTPO remained independent predictors of patellar osteosclerosis (Table 4). A more negative ΔPTS was associated with a higher likelihood of osteosclerosis (OR = 0.49, 95% CI = 0.34–0.71; *p* = 0.0002), and a smaller ΔFTPO (indicating greater femoral anteriorization) was likewise associated with osteosclerosis (OR = 0.78, 95% CI = 0.65–0.93, *p* = 0.0069). Age and sex were not significantly associated with the outcome.

**Table 4:** Multivariate logistic regression analysis for factors associated with patellar osteosclerosis.

Variable	*β* coefficient	Odds ratio (95% CI)	*p*
ΔPTS (°)	−0.73	0.49 (0.34–0.71)	0.0002
ΔFTPO (mm)	−0.25	0.78 (0.65–0.93)	0.0069
Age (y/o)	0.07	1.07 (0.93–1.23)	0.37
Sex (male)	1.42	4.15 (0.81–21.2)	0.088

*Note*: Δ indicates the difference between post‐operative and preoperative values (Post‐op minus Pre‐op).

Abbreviations: CI, confidence interval; FTPO, femorotibial posterior overhang; PTS, posterior tibial slope.

The combined multivariable model including both ΔPTS and ΔFTPO achieved an AUC of 0.93 (95% CI = 0.88–0.98), indicating excellent discriminative performance.

## DISCUSSION

The principal finding of this study is that post‐operative changes in sagittal alignment, specifically ΔPTS and ΔFTPO, were independently associated with the development of patellar osteosclerosis after TKA without patellar resurfacing. Both ΔPTS and ΔFTPO showed good discriminative ability for the presence of osteosclerosis, and a combined multivariable model including these two parameters demonstrated excellent performance. These results indicate that alterations in sagittal alignment, rather than absolute post‐operative values alone, may play a key role in the radiographic change observed in the patella following TKA.

Patellar osteosclerosis was observed in 27.7% of knees at one year post‐operatively. Although this incidence provides clinical context and was lower than that reported in some previous series of non‐resurfacing TKA [8], the more clinically relevant observation in the present study is that knees developing osteosclerosis exhibited a characteristic pattern of sagittal alignment change: greater preoperative PTS, post‐operative reduction in PTS, and increased relative femoral anteriorization reflected by smaller ΔFTPO. This pattern suggests that deviation from the patient's native sagittal alignment may be more important than achieving a uniform post‐operative alignment across patients. Regarding the underlying mechanism, we hypothesize that elevated PF joint stress induces adaptive remodelling of the subchondral bone. In accordance with Wolff's law and the mechanostat theory, this increased mechanical loading results in a localized increase in bone density, which manifests radiographically as osteosclerosis.

Regarding PTS, previous biomechanical and computational studies have shown that increasing PTS shifts the femorotibial contact point posteriorly and can reduce PF contact stress [11], while decreasing PTS increases PF loading [16]. The present findings align with this evidence: knees with osteosclerosis started with greater native PTS but experienced a post‐operative reduction, whereas the NOS group showed slight increases in PTS. Consequently, post‐operative PTS values were similar between groups, despite clear differences in ΔPTS. This supports the concept that reduction from native PTS, rather than an ‘abnormal’ post‐operative PTS value, may elevate PF stress by causing earlier PF engagement during flexion.

FTPO represents the anteroposterior relationship between the femur and tibia in the sagittal plane [14]. In this study, knees with osteosclerosis showed more posterior femoral positioning preoperatively and more anterior positioning post‐operatively, resulting in significantly smaller ΔFTPO values. Greater anteriorization may arise due to factors such as femoral component sizing, AP gap balancing, or posterior‐referencing techniques. From a biomechanical standpoint, anterior femoral positioning shortens the distance between the patella and the trochlea, potentially increasing PF contact stress—particularly when combined with reduced PTS. Although posterior femoral positioning has been associated with improved flexion and native‐like femorotibial mechanics [17], its influence on PF loading has been less documented. Our results highlight FTPO as a key sagittal parameter relevant to PF mechanics after TKA.

The positive correlation between ΔPTS and ΔFTPO indicates that a reduction in tibial slope tends to accompany anterior femoral translation. This interdependence is particularly relevant in BCS TKA designs. The Journey II BCS system used in this study employs a post‐cam mechanism to reproduce more physiological femoral rollback than conventional posterior‐stabilized designs [22]. Murata et al. reported smaller FTPO values in Journey II BCS compared with a conventional design, suggesting closer replication of native femorotibial positioning [14]. On the other hand, BCS‐type TKA has been reported to generate higher PF pressures than conventional designs [19]. In such mechanically constrained systems, reduced PTS may result in anterior translation of the femur relative to the tibia, leading to increased PF loading. The independent predictive ability of both ΔPTS and ΔFTPO in this study underscores the need for careful sagittal alignment control in BCS TKA.

Clinically, these findings emphasize the importance of patient‐specific sagittal alignment rather than uniform alignment targets. Even when post‐operative PTS is within an acceptable range, substantial reductions from a patient's native PTS may increase the risk of osteosclerosis. Similarly, excessive femoral anteriorization—as represented by smaller ΔFTPO—may be undesirable. Surgical strategies that preserve patient‐specific sagittal alignment, avoid excessive PTS reduction and minimize anterior femoral positioning may help mitigate PF overload, especially in TKA without patellar resurfacing.

Patellar management remains a topic of debate in TKA. In this study, patellar resurfacing was selected using PFA‐based criteria [20], and PFA itself was not associated with osteosclerosis, supporting the validity of this approach. However, the independent effects of ΔPTS and ΔFTPO suggest that sagittal alignment should also be considered when determining patellar treatment. In patients with steep preoperative PTS or sagittal profiles prone to substantial change with standard cuts, attention to sagittal alignment—and selective resurfacing in some cases—may be warranted.

This study has several limitations. First, its retrospective, single‐centre design may limit generalizability. Second, only one BCS implant system was evaluated, and results may not apply to other designs. Third, patellar osteosclerosis was used as an indirect surrogate for PF stress; direct pressure measurements and clinical symptoms such as anterior knee pain were not included. Although SPECT/CT findings from previous reports suggest that subchondral metabolic changes can correlate with PF symptoms [15, 24], the present study did not evaluate post‐operative AKP or patient‐reported outcomes, and therefore, the clinical relevance of osteosclerosis remains uncertain. Fourth, the follow‐up period was limited to 1 year, and the longer‐term implications of sagittal alignment changes and osteosclerotic findings remain unclear.

Additionally, although multivariable logistic models were employed, the limited sample size and number of events may have reduced the ability to detect weaker associations. Several factors known to influence PF loading—including patellar height, patellar thickness, femoral component rotational alignment and trochlear geometry, and body mass index—were not included in the adjustment model, and residual confounding cannot be ruled out. Furthermore, although all procedures were performed by a limited number of surgeons following standardized surgical techniques, surgeon‐specific factors may still have influenced the study outcomes.

Despite these limitations, this study provides radiographic evidence that changes in PTS and FTPO are independently associated with patellar osteosclerosis after TKA without patellar resurfacing. These sagittal alignment changes may contribute to increased PF stress and subsequent anterior knee pain. Surgeons should avoid excessive reduction in PTS and unintended anterior femoral positioning, particularly in BCS‐type implants and in knees treated without resurfacing. Future prospective studies, including direct PF pressure measurements, assessment of clinical symptoms and validation in other implant designs, are warranted.

## CONCLUSION

In TKA without patellar resurfacing, post‐operative reductions in PTS and relative femoral anteriorization, reflected by changes in FTPO, were independently associated with patellar osteosclerosis. These results highlight the importance of sagittal alignment changes—rather than absolute post‐operative values—in influencing patellar radiographic findings. Avoiding excessive PTS reduction or femoral anteriorization may help reduce PF overloading in non‐resurfaced TKA.

## AUTHOR CONTRIBUTIONS

All authors are responsible for the work described in this paper. All authors were involved in the conception, design and planning of the study. Shunsuke Utsumi, Takashi Aki and Yu Mori were involved in data measurement and analysis. All authors interpreted the study results, contributed to the critical review and approved the final manuscript.

## CONFLICT OF INTEREST STATEMENT

The authors declare no conflicts of interest.

## ETHICS STATEMENT

This retrospective study was conducted following the ethical standards outlined in the Declaration of Helsinki and was approved by the Institutional Review Board of Tohoku University Hospital (approval number 2022‐1‐066).

## Data Availability

The authors have nothing to report.
